# Audit of IV pantoprazole: pattern of administration and compliance with guideline in a teaching hospital

**DOI:** 10.1186/s40064-016-3428-2

**Published:** 2016-10-07

**Authors:** Mandana Moradi, Samaneh Raeesi, Zahra Sepehri

**Affiliations:** 1 Clinical Pharmacist, Zabol University of Medical Sciences, Zabol, Iran; 2 Student Research Committee, Zabol University of Medical Sciences, Zabol, Iran; 3 Internist, Internal Medicine Department, School of Medicine, Zabol University of Medical Sciences, Zabol, Iran

**Keywords:** IV pantoprazole, Drug use evaluation, Guideline, Audit

## Abstract

**Background:**

Drug use evaluation is a performance improvement method that evaluates medication-use processes. Medication studies are especially important for drugs with narrow therapeutic index, specific indication, high costs as well as for drugs with widespread use. Intravenous pantoprazole always ranked among the top 5 costly drugs in Amir-al-Momenin Hospital. Considering the fact that widespread and inappropriate use of this drug is considered as a concern in hospitals all over the world rather than being a regional problem, we decided to establish a guideline for intravenous (IV) pantoprazole in our hospital and evaluated the pattern of its administration both before and after establishing the guideline.

**Methods:**

This is an experimental study (clinical trial) performed at the Amir-al-Momenin Hospital, on 400 randomly selected patients receiving IV pantoprazole (bolus or infusion) during a 6-month period (3 months before and 3 months after establishing guideline). We used predesigned data collection forms to collect related information. We used SPSS Ver. 18 for statistical analysis.

**Results:**

Our results showed that the established guideline could significantly reduce the rate (P = 0.00) and cost (301,289,000 Rials or 8608 USD in 3 months study period after establishing guideline) of IV pantoprazole administration, but failed to rectify indications and dosage of its administration at the same rate. Stress ulcer prophylaxis was the most frequent approved indication for IV pantoprazole administration in our study population. We also observed that the rate of commitment to the guideline decreased by the time passed from its establishment.

**Conclusion:**

We concluded that although establishing guideline was successful in reducing the overall rate of IV pantoprazole administration and its related costs, different contributing factors halted its effect on correcting the prescribed dosage and indications, especially as the time gaps from guideline establishment. This fact magnifies the importance of continuous educations of prescribers about the importance of evidence based practice and need for and implementing a powerful executive supervisory in our hospital.

## Background

Drug use evaluation (DUE) studies are systematic methods of obtaining information to identify drug related problems, ranging from doctors’ orders to nurses’ administration, with the aim of optimization of drug use patterns especially in hospitals. DUEs are important contributors of rational drug use (Chomsky 2012). Based on World Health Organization (WHO) definition “Drug use evaluation (DUE) is a system of ongoing, systematic, criteria-based evaluation of drug use that will help ensure that medicines are used appropriately (at the individual patient level)” (Hepler and Strand 1990). These types of studies are especially important for drugs with narrow therapeutic index, specific indications, or for expensive or widely administered drugs (Moore et al. 1997).

Pantoprazole is a proton pomp inhibitor (PPIs) with both oral and intravenous (IV) dosage forms. Results of different studies showed that despite more rapid onset of action in IV pantoprazole, both dosage forms (oral and intravenous) can reduce gastric acid secretion to the same extent (O 2015). The decision to select an appropriate dosage form depends on several factors; such as patient’s ability to take oral medication, patient’s hemodynamic status as well as intestinal permeability and absorptive capacity (Pang and Graham 2010). These factors often should be considered especially in critically ill patients when pantoprazole is indicated for either treating an acid secreting disorder or prophylaxis of stress related mucosal injury (Pang and Graham 2010).

Intravenous pantoprazole was first marketed in Canada in 1999, since then it has been used for treatment of different pathological conditions in which rapid reduction of gastric acid is required (Kaplan et al. 2005).

Currently it is the only IV proton-pump inhibitor commercially available in North America as well as our country, so it is not surprising that this drug is widely used in hospitals. Inappropriate and unsupervised use of IV pantoprazole can lead to unwanted consequences such as increased treatment cost, adverse effects related to injection and increasing the incidence of nosocomial pneumonia, spontaneous bacterial peritonitis (SBP) and *Clostridium difficile* infections (CDI) (Pang and Graham 2010).

As IV pantoprazole always ranked among the 5 top high costs drugs in our hospital, also, considering the significant difference between the price of IV pantoprazole with other therapeutic options (capsules of pantoprazole, parenteral ranitidine) as well as potential complications associated with unsupervised administration of parenteral formulations and its indirect cost, we established a guideline and evaluated the pattern of its administration in our hospital, both before and after guideline establishment. Our aim was to optimize drug administration, decrease our therapeutic cost and measure the effectiveness of guideline establishment as a potential solution.

## Methods

After being approved by ethics committee of Zabol University of Medical Sciences, about 400 patients receiving IV pantoprazole (bolus or infusion) during a 6-month period, both before (as control group) and after (as intervention group) establishing a medical guideline for administration of IV pantoprazole, were randomly selected (from total of 1150 patients who were prescribed IV pantoprazole in this period). This guideline was prepared by our pharmacy department and took the approval on January 2015 by the hospital Drug and Therapeutic (D&T) Committee to be implemented. The guideline was mailed to all physicians who work in our hospital with an official cover letter signed by the hospital manager about the necessity of commitment to the approved guideline. An educational class was also conducted by the pharmacy department for doctors and nurses, separately.

Patients were randomly selected from the inpatient pharmacy computer database at the Amir-al-Momenin Hospital (affiliated to Zabol University of Medical Sciences). Each patient chart was manually reviewed by the investigator, using predesigned data collection forms. The following information was abstracted:

Patients demographic data, primary diagnosis, history of previous use of PPIs, past medical history especially history of GI disease, indication for IV pantoprazole administration, dose and duration of IV pantoprazole administration, prescribing service, type of patient’s diet [oral or non per oral (NPO)], other medications prescribed during IV pantoprazole administration, duplication therapy (described as taking two or more drugs with the same mechanism of action or the probable effect on one organ)

In patients admitted with diagnoses of upper GI bleeding, the following extra information was also recorded: symptoms indicative of upper GI bleeding and their onset, endoscopic results, recent myocardial infarction or other significant co-morbidities making endoscopy are potentially dangerous (Facts and Comparision 2016; Guilford 1990).

### A: Definition of appropriate indication

 The appropriate indications for IV pantoprazole based on our approved administration guideline were as follows:

IV pantoprazole was considered to be indicative when patient was NPO (nothing per oral) and manifested with at least one of the following conditions:

Erosive esophagitis associated with gastrointestinal reflux disease (GERD) (Barkun et al. 2010).

Pathologic hyper secretion associated with Zollinger–Ellison syndrome (Barkun et al. 2010).

Upper gastrointestinal bleeding (UGIB) and prevention of re-bleeding (Barkun et al. 2010; Buckley et al. 2015).

Stress ulcer prophylaxis (SUP) (Friedman et al. 2001).

In patients who could tolerate oral medication and those who are candidate of PPI therapy based on our guideline, we introduced oral pantoprazole as an alternative treatment.

### B: Definition of appropriate dosing regimen

Erosive esophagitis associated with GERD: 40 mg once daily for 7–10 days (American Pharmacist Association 2014).

Hyper secretary disorders (including Zollinger–Ellison): 80 mg every 12 h; adjust dose based on acid output measurements; 160–240 mg daily in divided doses were used for a limited period (Barkun et al. 2010).

*UGIB* an initial 80-mg bolus followed by an 8 mg/h infusion for 72 h in upper gastrointestinal hemorrhage. If re-bleeding occurred, diagnosed on clinical and/or endoscopic grounds, the patient is allowed to receive IV PPI for an additional 72 h (Barkun et al. 2010).

*SUP* 40 mg once daily (Cohen 2013).

### C: Costs

The pharmaceutical cost was calculated based on the national marketing cost of 40-mg vial of pantoprazole; 120000 Rials (3.5 USD). Costs were calculated for the entire treatment period. Indirect costs as the price of syringe and injecting the IV dosage form were not included.

### D: Statistical analysis

Data were entered into the Statistical Package for Social Sciences (SPSS) version 18. Differences in appropriate prescription during the pre and post intervention periods were tested using Chi square. A *p* value of <0.05 was statistically considered as significant.

## Results

### Part 1: Extent of the problem

Overall 846 patients were treated with IV pantoprazole during the 3 months period before guideline establishment (from October 2014 until December 2014) and 200 cases were randomly selected to include in this study.

The mean age of our study population was 54 years; whose demographic data are presented in Table 1. Details of doctor’s specialty that commenced the IV pantoprazole therapy in these patients were as follows: General physicians 7 %, Internists 37 %, Surgeons 6.5 %, Cardiologists 33.5 % and other specialties 16.5 %.

**Table 1:** Characteristics of patients receiving IV pantoprazole before and after of guideline establishment

Characteristic	Pre intervention Number (%)	Post intervention Number (%)	P value^b^
Male	85 (42.5)	118 (59.0)	0.01
Female	115 (57.5)	82 (41.0)	
Age (years)			
Mean ± SD^b^	54.56 ± 1.273	48.09 ± 1.493	
<20	5 (2.5)	27 (13.5)	0.01
20–30	15 (7.5)	21 (10.5)	
30–40	26 (13)	22 (11)	
40–50	29 (14.5)	37 (18.5)	
50–60	53 (26.5)	39 (19.5)	
60–70	30 (15)	21 (10.5)	
>70	21 (10.5)	33 (16.5)	
NPO	54 (27.0)	99 (49.5)	0.00
PO	146 (73.0)	101 (50.5)	
History of PPI administration	59 (29.5)	37 (18.5)	0.33
History of GI disease	75 (37.0)	52 (26.5)	0.05
Death	1 (0.5)	1 (0.5)	1.00

^a^ *SD* standard deviation

^b^Calculated with Chi square test that <0.05 was considered as statistically significant difference

Our results showed that the majority (33.5 %) of the patients were admitted in CCU followed by internal disease ward and ICU, with 24.5 and 13 % of patients respectively.

A total of 92 (46.0 %) patients had at least one co-morbidity at the time of admission among which cardiovascular diseases [n = 31 (15.5 %)] were the most prevalent co-morbidity followed by diabetes [n = 27 (13.5 %)] and hypertension [n = 14 (7.0 %)]. Co-medications are described in details in Table 2.

**Table 2:** Drug prescribed with IV pantoprazole

Pharmacologic category	Number (%)		P value
	Before guideline	After guideline	
Cardiovascular drugs	81 (40.5)	43 (21.5)	0.00
Sedative/narcotics	14 (7)	4 (2)	
Antibiotics	33 (16.5)	42 (21)	
Gastrointestinal drugs	17 (8.5)	64 (32)	
Anticoagulants	11 (5.5)	–	
Lipid lowering agents	5 (2.5)	6 (3)	
Anti-emetics	8 (4)	–	
Anti-convulsant	2 (1)	–	
Respiratory drugs	5 (2.5)	27 (13.5)	
Vitamins and minerals	3 (1.5)	1 (0.5)	
Corticosteroids	1 (0.5)	2 (1)	
Antidepressants	2 (1)	1 (0.5)	
Others	18 (9)	10 (5)	

### Part 2: Effect of multidisciplinary intervention

Total of 304 patients received IV pantoprazole during the 3 month-study period after guideline establishment (from February 2015 until April 2015) and 200 cases were randomly selected to enter this study.

The mean age of these patients was 48.09 years (Table 1). The pattern of prescribers who initiated IV pantoprazole was as follows: General physicians 6 %, Internists 45.5 %, Surgeons 18 %, Cardiologist 15 % and other specialties 15.5 %. Ward distribution changed as follows: internal ward ranked first with 28 % of patients followed by ICU and CCU with 24 and 13 % of patients showing statistically significant difference between these two study periods.

A total of 62 (31 %) patients had some sort of co-morbidities on admission, which hypertension [n = 19 (9.5 %)] ranked first followed by cardiovascular disease [n = 13 (6.5 %)] and diabetes [n = 11 (5.5 %)]. Other base line characteristics of patients, after guideline establishment, are described in Table 1. Drugs prescribed with IV pantoprazole, are described in Table 2 in details.

During our study period, we found a few nursing mistakes in drug administration that could interfere with our results; 4 patients who were prescribed capsules of pantoprazole received IV dosage form by mistake and 5 patients were taken wrong dose compared to the ordered dosage regime by the doctors.

### Part 3: Appropriateness of intravenous pantoprazole administration

The overall number of IV pantoprazole administration and total number of patients who received this drug were 2510 and 304 respectively after guideline establishment compared to 5385 and 846 before implementing guideline. Calculated P values (P = 0.00 for both factors) show statistically differences between pre and post guideline establishment period.

Regarding the indications of IV pantoprazole administration, our results showed that guideline establishment could significantly reduce the frequency of cases with disapproved indications which, abdominal pain relief and UGIB prophylaxis(in patients receiving Aspirin and/or Clopidogrel) were the first and second most frequent disapproved indications (P = 0.00) (Table 3).

**Table 3:** Indications of IV pantoprazole administration before and after guideline establishment

Indications	Before guideline (n = 200), (%)	After guideline (n = 200), (%)	P value^b^
NPO patients with erosive esophagitis (EE) with GERD^a^	2 (1.0)	5 (2.5)	0.00
Prophylaxis of rebleeding in NPO patients^a^	7 (3.5)	16 (8.0)	
Stress ulcer prophylaxis (SUP)in NPO patients^a^	17 (8.5)	47 (23.5)	
PUD^c^	2 (1.0)	2 (1.0)	
GIB prophylaxis in patients receiving anti coagulation therapy	38 (19.0)	15 (7.5)	
Abdominal pain	50 (25.0)	32 (16.0)	
Undetermined indication^d^	86 (42.0)	83 (41.5)	

^a^Indications approved for iv pantoprazole administration by the guideline

^b^Calculated with Chi square test that < 0.05 was considered as statistically significant difference

^c^ *PUD* peptic ulcer disease

^d^Based on the contents of the patients’s files we could not find any justification for the administration of the drug

In patients receiving IV pantoprazole, the frequency of approved indications was 11 % in the first month after guideline approval then increased to 12.5 % in the second month and dropped to 10.5 % in the third months of our study.

### Stress ulcer prophylaxis

Stress ulcer prophylaxis was the most frequent approved indication for IV pantoprazole administration in our study population, both before and after guideline establishment. Recommended risk factors for stress ulcer prophylaxis by American Society of Health system Pharmacists (ASHP) are mentioned in Table 4. Our results indicated that the frequency of presence of these risk factors in patients received iv pantoprazole for SUP, significantly increased after guideline establishment (P = 0.00) (Table 4), and the most frequent risk factor in our study population was mechanical ventilation for more than 48 h.

**Table 4:** Risk factors for stress ulcer prophylaxis in patients treated for this indication

Risk factors	Before guideline N (%)	After guideline N (%)	P value^c^
Coagulopathy^a^	0 (0)	1 (2.1)	0.00
Mechanical ventilation for >48 h	11 (64.7)	24 (51.1)	
History of GI ulceration or bleeding within the past year	1 (4.3)	2 (4.2)	
Traumatic brain injury, traumatic spinal cord injury	1 (5.9)	9 (19.1)	
Burn injury	1 (5.9)	1 (2.1)	
Two or more of minor criteria^b^	3 (17.6)	10 (21.4)	
Total	17 (100.0)	47 (100.0)	

^a^Coagulopathy defined as a platelet count <50,000 per m^3^, an International Normalized Ratio (INR) >1.5, or a partial thromboplastintime (PTT) >2 times the control value

^b^Minor criteria: sepsis, an intensive care unit (ICU stay) >1 week, occult GI bleeding for ≥6 days, or glucocorticoid therapy (more than 250 mg hydrocortisone or the equivalent

^c^Calculated with Chi square test that <0.05 was considered as statistically significant difference

### Upper gastrointestinal bleeding

It was observed that 16 patients diagnosed with UGIB were admitted and commenced on IV pantoprazole, after guideline establishment, compared to 7 patients before guideline approval (P = 0.053). All these patients manifested common symptom of UGIB as melena (black tarry stool), hematemesis (either red blood or coffee-ground emesis) and hematochezia (red or maroon blood in the stool).

Endoscopy was performed in 42.9 % of these patients before guideline establishment. Although, this rate increased to 50 % after approving guideline, but this difference was not statistically significant (P = 0.871). We also found that IV pantoprazole was discontinued in all patients with no sign of bleeding or high risk stigma in endoscopic results after guideline establishment, while before guideline approval drug therapy was continued regardless of the results of endoscopy. Details are depicted in algorithm 1.
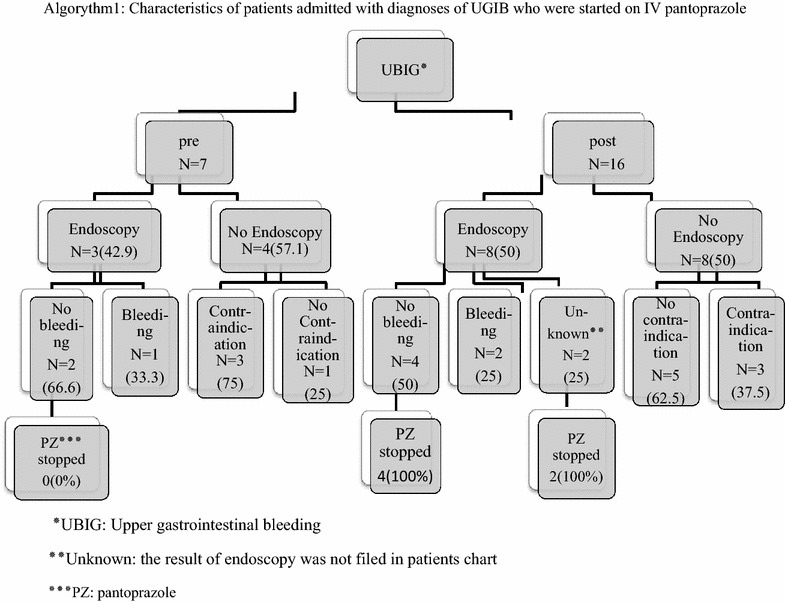


### Administered therapeutic regimen (Dose & Duration)

The most commonly prescribed dosage regimen in our study population was 40 mg two times a day. We observed 56.25 % of patients received recommended dose for the mentioned indication after guideline establishment, compared to 28.6 % of patients before guideline (P = 0.032). We also found that most inappropriate prescribed dosage regimen either pre or post guideline establishment, were higher than recommended amount (Table 5).

**Table 5:** Intravenous pantoprazole administered regimen before and after guideline establishment

Diagnoses	Dose		
	80 mg IV, then 8 mg/h	40 mg IV bid	Duration (day) Mean ± SD
UGIB Pre Post p-value	28.6 % 56.25 % 0.032	71.4 % 43.75 % 0.558	2.86 ± 1.464 2.75 ± 2.517
SUP Pre Post p-value	0 0 1.0	100 % 100 % 1.0	4.47 ± 3.970 5.13 ± 3.621
EE^a^ with GERD Pre Post p-value	0 0 1.0	100 % 100 % 1.0	0.5 ± 0.707 2.00 ± 0.707

^a^Erosive esophagitis

### Cost analysis

There was statistically significant pharmaceutical cost difference between pre and the post intervention (P = 0.00). The costs of IV pantoprazole therapy in 3 months period before guideline establishment was 646166000 Rials (18462 USD) compared to 344877000 Rials (9853 USD) in the post intervention period, with a potential cost savings of 301289000 Rials (8608 USD).

## Discussion

The results of this study indicated that although establishment of administration guideline for this drug, could significantly reduce the overall frequency of IV pantoprazole administration and its total costs, but could not correct indications and dosage of prescribed IV pantoprazole effectively. Comparison of our results with other similar trials, for example those conducted in Canada (Kaplan et al. 2005), showed that the rate of inappropriate administration of this drug did not significantly decrease after guideline establishment in our study as well as Canadian study. However our achievement rate in decreasing administration of pantoprazole through guideline establishment was even lower comparing with Canadian study (appropriate IV pantoprazole administration rate of 29 % in Canada after guideline establishment compare to about 11 % in our study). It justifies further investigation about the potential causes of this failure in our hospital.

As we discussed before, intravenous pantoprazole was widely used with disapproved indications in our hospital like what has been reported from other parts of the world, which unexplained abdominal pain was the most prevalent mentioned indication in patient’s charts of this group of patients. Unfortunately, the exact cause of abdominal pain was not thoroughly investigated in the majority of patients. Besides, doctors commonly preferred parenteral formulation over oral dosage form maybe due to the unrealistic fear of under–treatment with oral formulation of this drug or even H2 blockers. These two facts lead to overuse of intravenous pantoprazole administration and its overall costs (Lai et al. 2014).

Prevention of gastrointestinal (GI) bleeding in patients taking Clopidogrel and/or ASA was the second most prevalent unapproved indication for prescribing intravenous pantoprazole in our study population. American heart association and American College of Cardiology in 2007 suggested gastric acid suppression for the prevention of GI bleeding in patients taking antiplatelet therapy,^1^ but since PPIS have the potential to inhibit cyp2c19isoenzymes, they may interact with Clopidogrel effects. This fact makes H2 antagonist first choice for this indication (Skledar and Culley 2005).

In this study, all patients admitted with diagnoses of upper gastrointestinal bleeding (UGIB) initially received IV pantoprazole recommended by the guideline. But similar to some other studies, before the guideline establishment, doctors ignored to discontinue intravenous pantoprazole in whom endoscopic findings were not indicative of bleeding (Kaplan et al. 2005; Cornish et al. 2002; Jutabha et al. 2015; Wilkins et al. 2012). This contributes to increase the rate of inappropriate use of this drug. This audit revealed that upper gastrointestinal endoscopy (UGIE) was performed in only 50 % of patients with suspected UGIB post intervention (vs 42.9 pre intervention). The data obtained reported from many Canadian centers, similarly revealed that in patients admitted with diagnoses of UGIB, IV pantoprazole is often commenced before the results of endoscopy is available, and continued regardless of endoscopic findings (Leape et al. 1999). Also another study performed in Colombia, showed that 57 % of patients presenting UGIB received IV pantoprazole before the result of endoscopy is available (Tsoi et al. 2013). These findings are contrary to some other studies which showed uses of IV pantoprazole were based on the result of UGIE (Alsultan et al. 2010).

Evaluation of the outcome of the patients was not one of our goals in our study; hence, we did not observe any gastrointestinal complication such as bleeding during the study period. However, we indicated in our study that most of our patients were prescribed pantoprazole without scientific indication.

Admittedly, in this study we unfortunately observed that as time passes from guideline establishment, the rate of its effectiveness decreases continuously. We believe that the lack of continuous supervisory interventions by our pharmacy department, for example because of the shortage of trained active pharmacist in the hospital is an important contributor.

Finally, we believe that the major limitation of this study was that we merely relied on the medical records of patients. Consequently, any limitation in documenting process of patient’s charts could interfere with our final results. Another limitation was that we focused on IV pantoprazole instead of pantoprazole. Other studies are recommended to evaluate pantoprazole usage in the educational hospitals.

## Conclusion

We conclude that although guideline establishment is an effective tool for rational drug use in hospitals, it does not necessarily guarantee appropriate drug administration. The promotion of prescriber knowledge about evidence-based medicine and update national and international guidelines on drug administration through continuous educational programs as well as implementing powerful executive constitutions in hospitals, are other important contributing factors that can influence the rate of guideline achievement.

 At the end, some suggestions based on our experience in our hospital, for increasing compliance to the guideline there are two ways. First, in our hospital, the cost of the inappropriate prescription of the expensive drugs was charged on the prescribers for a while. It was effective in decreasing the costs. It could be continued until the complete establishment of the guideline. Second, we designed a form for expensive drugs such as pantoprazole in which the physicians should sign by mentioning the indication for the prescription of drug. It was effective in improving the appropriate prescription of the drugs (it is under study). This might be effective and should be evaluated in future studies.

## Abbreviations

APT: antiplatelet therapy
ASA: acetyl salicylic acid
ASHP: American Society of Health System Pharmacists
CDI: clostridium difficile infections
DUE: drug use evaluation
D&T: drug and therapeutic
EE: erosive esophagitis
GERD: gastroesophageal reflux disease
GI: gastrointestinal
ICU: intensive care unit
INR: International Normalized Ratio
IV: intravenous
NPO: nothing by mouth
PPI: proton pump inhibitor
PTT: partial thromboplastin time
PZ: pantoprazole
PUD: peptic ulcer disease
SBP: spontaneous bacterial peritonitis
SUP: stress ulcer prophylaxis
SPSS: statistical package for social sciences
UGIB: upper gastrointestinal bleeding
UGIE: upper gastrointestinal endoscopy
UK: United Kingdom
USA: United States of America
WHO: World Health Organization

## Footnotes

1 Antiplatelet therapy (APT) has been found to reduce the risk of cardiac death, myocardial infarction, stroke, and stent thrombosis following acute coronary syndrome and percutaneous coronary intervention. However, this therapy has also been shown to increase the risk of gastrointestinal (GI) bleeding as high as twofold, especially in patients with multiple risk factors (see Morneau et al. 2014).
